# Cdk1-mediated threonine phosphorylation of Sam68 modulates its RNA binding, alternative splicing activity and cellular functions

**DOI:** 10.1093/nar/gkac1181

**Published:** 2022-12-20

**Authors:** Idir Malki, Inara Liepina, Nora Kogelnik, Hollie Watmuff, Sue Robinson, Adam Lightfoot, Oksana Gonchar, Andrew Bottrill, Andrew M Fry, Cyril Dominguez

**Affiliations:** The Leicester Institute of Structural and Chemical Biology and Department of Molecular and Cell Biology, University of Leicester, Leicester LE1 7RH, UK; The Leicester Institute of Structural and Chemical Biology and Department of Molecular and Cell Biology, University of Leicester, Leicester LE1 7RH, UK; The Leicester Institute of Structural and Chemical Biology and Department of Molecular and Cell Biology, University of Leicester, Leicester LE1 7RH, UK; The Leicester Institute of Structural and Chemical Biology and Department of Molecular and Cell Biology, University of Leicester, Leicester LE1 7RH, UK; The Leicester Institute of Structural and Chemical Biology and Department of Molecular and Cell Biology, University of Leicester, Leicester LE1 7RH, UK; The Leicester Institute of Structural and Chemical Biology and Department of Molecular and Cell Biology, University of Leicester, Leicester LE1 7RH, UK; The Leicester Institute of Structural and Chemical Biology and Department of Molecular and Cell Biology, University of Leicester, Leicester LE1 7RH, UK; Proteomics RTP, School of Life Sciences, University of Warwick, Gibbet Hill Road, Coventry CV4 7AL, UK; Department of Molecular and Cell Biology, University of Leicester, Leicester LE1 7RH, UK; The Leicester Institute of Structural and Chemical Biology and Department of Molecular and Cell Biology, University of Leicester, Leicester LE1 7RH, UK

## Abstract

Sam68, also known as KHDRBS1, is a member of the STAR family of proteins that directly link signal transduction with post-transcriptional gene regulation. Sam68 controls the alternative splicing of many oncogenic proteins and its role is modulated by post-translational modifications, including serine/threonine phosphorylation, that differ at various stages of the cell cycle. However, the molecular basis and mechanisms of these modulations remain largely unknown. Here, we combined mass spectrometry, nuclear magnetic resonance spectroscopy and cell biology techniques to provide a comprehensive post-translational modification mapping of Sam68 at different stages of the cell cycle in HEK293 and HCT116 cells. We established that Sam68 is specifically phosphorylated at T33 and T317 by Cdk1, and demonstrated that these phosphorylation events reduce the binding of Sam68 to RNA, control its cellular localization and reduce its alternative splicing activity, leading to a reduction in the induction of apoptosis and an increase in the proliferation of HCT116 cells.

## INTRODUCTION

Alternative splicing of pre-mRNA is a highly regulated mechanism that allows for the generation of proteomic diversity from a limited number of human genes (1). Cell signalling plays a major role in the regulation of alternative splicing (2,3), and splicing factor mutations or copy number variations are associated with many genetic diseases, including cancer (4,5).

A typical example is the splicing factor Sam68 (Src-associated protein during mitosis of 68 kDa), also known as KHDRBS1 (KH domain-containing, RNA-binding, signal transduction-associated protein 1), that controls the alternative splicing of many pre-mRNAs coding for oncogenic proteins, such as cyclin D1, CD44 and Bcl-x (6–8). Sam68 plays important roles in cell cycle regulation, apoptosis and viral replication, through its functions in various aspects of gene expression, including transcription, pre-mRNA splicing and RNA export (9,10). Sam68 is also an oncogene (10,11): high expression of Sam68 correlates with poor prognosis in many cancers, including prostate and colon cancers (12,13), while depletion of Sam68 decreases cell migration in HeLa cells (14), and inhibits proliferation and tumour progression in prostate and breast cancers (12,15). Importantly, Sam68’s cellular functions are regulated by cell signalling through post-translational modifications (PTMs), providing a direct link between cell signalling and post-transcriptional gene regulation (9,10).

Sam68 belongs to the STAR (Signal Transduction and Activation of RNA) family of proteins (16,17) and contains a central QUA1/KH domain that is responsible for its homodimerization and RNA binding. This domain is flanked by N- and C-terminal regulatory regions, such as a tyrosine-rich region (YY) targeted by tyrosine kinases, proline-rich (P) regions responsible for binding SH3 and WW domain-containing proteins, Arg/Gly (RG)-rich regions targeted by arginine methyltransferases, and a nuclear localization signal (NLS). We have recently revealed the structural basis of RNA recognition and dimerization by the QUA1/KH domain and proposed a model for the role of Sam68 in alternative splicing whereby Sam68 dimers loop out regions of its target pre-mRNAs to induce either exon inclusion or skipping (18).

It is well known that the cellular functions of Sam68 are controlled by PTMs, including tyrosine, serine and threonine phosphorylation by the Src kinase family (19,20), Erk1 (7), Cdk1 (21) and Nek2 (22), arginine methylation by PRMT1 (23), sumoylation by PIAS1 (24), lysine acetylation by CBP (25) and deacetylation by HDAC6 (26). For example, phosphorylation of Sam68 by Src-like kinases enhances the production of the oncogenic isoform of Bcl-x (8).

While tyrosine phosphorylation and arginine methylation of Sam68 have been well documented, serine/threonine (S/T) phosphorylation of Sam68 remains largely unexplored (27). Previous research in HeLa and NIH3T3 cells showed that serine residues of Sam68 are phosphorylated throughout the cell cycle but that threonine phosphorylation occurs only during mitosis (21). Further analysis demonstrated that Sam68 threonine phosphorylation is abrogated in cell extracts treated with a CDK1 inhibitor and that CDK1 is able to phosphorylate Sam68 threonines *in vitro* (21). Mutation of a putative Cdk1 target site (T317) reduced the levels of murine Sam68 phosphorylation by Cdk1 *in vitro* by 30%, suggesting that this is likely to be one site of phosphorylation but that at least one other phosphorylation site is present in Sam68. Precisely which residues of Sam68 are phosphorylated by Cdk1 and the functional consequences of these phosphorylation events remain unknown. Later, Sam68 was shown to be phosphorylated by Erk1 in EL4 and SW480 cells (7). Erk1-mediated phosphorylation of Sam68 stimulated the activity of Sam68 towards CD44 exon 5 inclusion (7), *Cyclin D1b* isoform production (6) and SRSF1 nonsense mRNA decay (28). However, the mechanisms of this stimulation are unclear since it has also been shown that Erk1-mediated phosphorylation of Sam68 reduced its RNA binding ability (29). A triple mutant, S58A/T71A/T84A, reduced the stimulation of CD44 exon v5 inclusion while single mutants did not, suggesting that the phosphorylation of at least one of these three residues is important for splicing stimulation (7). However, there is no direct evidence that these three residues are phosphorylated by Erk1. Furthermore, this study was done on murine Sam68, and T71 is not conserved in human Sam68. Sam68 was also shown to be phosphorylated at S20 following depolarization of neuronal cells, possibly by calcium/calmodulin-dependent protein kinase (CAMK) IV, leading to alterations in the splicing of Neurexin 1 (30), but it remains unclear whether this phosphorylation event is restricted to neuronal cells or is also present in other cell types. Finally, Sam68 has been recently shown to be phosphorylated by Nek2 in breast cancer cell lines and, like Erk1, Nek2-mediated phosphorylation of Sam68 stimulated its activity towards CD44 exon 5 inclusion (22). Using a truncated version of Sam68, its was shown that both the N- and C-terminal regions of Sam68 are phosphorylated by Nek2, but precisely which serines and threonines were phosphorylated remains unknown (22).

Here we used mass spectrometry (MS) to map the serine and threonine residues of Sam68 phosphorylated at different stages of the cell cycle in HEK293 and HCT116 cells. In addition, we have used nuclear magnetic resonance (NMR) spectroscopy to characterize the N- and C-terminal intrinsically disordered regions (IDRs) of Sam68 and the specific phosphorylation of Sam68 at T33 and T317 by Cdk1. We demonstrate that their phosphorylation not only reduces Sam68’s ability to bind RNA and regulate splicing, but also modulates its cellular localization and functions in apoptosis and proliferation. We propose a model whereby the intrinsically disordered regulatory regions of Sam68 anchor the protein to its target pre-mRNAs, thus increasing its affinity for RNA. However, during mitosis, phosphorylation of Sam68 by Cdk1 would reduce its affinity for RNA, which in turn would favour competing splicing factors to bind the pre-mRNA, resulting in switches in RNA splicing patterns.

## MATERIALS AND METHODS

### Protein and RNA constructs

Sam68 constructs were cloned by the University of Leicester cloning service (Protex, https://le.ac.uk/mcb/facilities-and-technologies/protex). Full-length His-tag/Flag-tag Sam68 was cloned into the pLEICS-12 vector, full-length green fluorescent protein (GFP)–Sam68 into the pLEICS-25 vector, His-Tag Sam68 N-terminal region (amino acids 1–96) into the pLEICS-01 vector and glutathione *S*-transferase (GST)–Sam68 C-terminal region (267–368) into pGEX-6P-2 (https://www.genscript.com/express-cloning-vector-list.html). Sam68 C-terminal cDNA was synthesized with optimized codon composition for bacterial expression (Genscript).

RNA oligonucleotides were purchased from Dharmacon, Horizon Discovery.

Bclx and CD44-v5 minigenes were cloned in the EGFP-C1 plasmid following the cytomegalovirus (CMV) promoter using ExoIII (NEB) enzyme following the manufacturer’s instructions. The CD44 minigene was cloned in two steps. Firstly, a DNA sequence comprising β-globin exon 2, intron 2 and exon 3 was cloned in AgeI and HindIII restriction sites of the EGFP-C1 plasmid using primers Bg-AgeI-Fwd (agatccgctagcgctACCGGTGGGCTGCTGGTTGTCTACCCATGG) and Bg-HindIII-Rev (aattcgaacttgagcGAATTCAACTTACCTGCCAAAATGATGAGAC). Secondly, CD44 variable exon 5 (v5, 117 bp long) with flanking intron sequences (238 bp upstream and 270 bp downstream) was polymerase chain reaction (PCR) amplified from genomic DNA using primers CD44-Fwd (AAATTCATGTTATATGGTCGACAGCCAACAGCCCTACAAATGTTAG) and CD44-Rev (AACATGGTTAGCAGAGTCGACACCCTTAGGAACCATTAACAC), and cloned into the SalI restriction site positioned in the β-globin intron 2. The Bcl-x minigene (31) was PCR amplified using primers Bcl-X-Fwd (TAGTGAACCGTCAGATCCGCTAGCGCTACCGGTGGGAGGTGATCCCCATGGCAG) and Bcl-X-Rev (GTCGACTGCAGAATTCGAAGCTTACTTACCTGGCCACAGTCAT), and cloned in AgeI and HindIII sites of the EGFP-C1 plasmid using ExoIII enzyme.

### Bacterial expression of Sam68 N- and C-terminal regions

The expression vectors (pLEICS-01 and pGEX-6P-2) were transformed into *Escherichia coli* Rosetta(DE3) cells. Cells were grown at 37°C to an absorbance of 0.8 and incubated with 0.5 mM isopropyl-β-d-thiogalactopyranoside (IPTG) at 20°C overnight. The His-Tag Sam68 N-terminal region was purified using a nickel affinity chromatography column. The His-tag was removed by incubating the protein with tobacco etch virus (TEV) protease in the presence of 0.5 mM EDTA and 1 mM ditiothreitol (DTT) at 15°C overnight. The protein sample was buffer exchanged using a PD-10 column (G25 resin, cut-off of 7 kDa; GE Healtcare) against 50 mM sodium phosphate buffer pH 7.6, 150 mM NaCl and then loaded on a nickel affinity chromatography column. The cleaved protein was then recovered in the flowthrough. The GST–Sam68 C-terminal region was first purified using a glutathione affinity column. The eluted protein was incubated with PreScission protease at 4°C overnight. The cleaved protein was then purified by a Superdex 75 size exclusion column using 50 mM sodium phosphate buffer pH 7.0, 150 mM NaCl as running buffer.

All the purification steps were performed in the presence of complete protease inhibitor cocktail (Roche). Uniformly ^15^N-, ^13^C-labelled proteins were expressed in M9 medium containing 1 g of ^15^NH_4_Cl and 2 g of [^13^C]glucose per litre of culture as the sole source of carbon and nitrogen.

### Site-directed mutagenesis

Site-directed mutagenesis was carried out using extension PCR with back-to-back oriented primers, with the site of mutation positioned at the beginning of the forward primer. PCR was carried out and the linear product of the PCR was purified, ligated and transformed into DH5α. Colonies were screened and verified by Sanger sequencing.

### Cell culture

HEK293T and HCT116 cells were grown in Dulbecco's modified Eagle’s medium (DMEM) supplemented with GlutaMAX™ (Gibco), 10% foetal bovine serum (Gibco), 1% v/v penicillin and streptomycin (Gibco) in a 5% CO_2_ incubator.

### Transfection

HCT116 cells were transfected using JetPrime(Polyplus) and HEK293 using Fugene (Promega) using a standard protocol. Cells were passaged 24 h before transfection. A total of 1.5–3 μg of DNA was used to transfect 0.2 × 10^6^ passaged cells. Transfection efficiency was assessed by visualization of the GFP signal and western blot analysis of GFP–Sam68.

### Mass spectrometry

Cells were passaged 24 h before transfection with Flag-Sam68. For unsynchronized samples, cells were harvested 48 h after transfection. For MS analysis at different stages of the cell cycle, cells were pre-synchronized with 2 mM thymidine 8 h after transfection, and were released from the arrest by three washes with phosphate-buffered saline (PBS) followed by addition of fresh medium 16 h after thymine addition. For mitotic samples, cells were synchronized by addition of 0.5 μg/ml nocodazole 8 h after release, and harvested 16 h after nocodazole addition. For G_1_ samples, cells were synchronized by addition of 1 μg/ml nocodazole 8 h after release from thymidine and then washed three times with PBS followed by addition of fresh medium and incubation for 6 h prior to harvesting. For S phase samples, cells were synchronized by addition of 1 mM hydroxyurea 8 h after release from thymidine and then washed three times with PBS followed by addition of fresh medium and incubation for 3 h prior to harvesting. Cells were lysed with RIPA buffer, and FLAG-Sam68 was pulled down using M2 FLAG beads. Purified FLAG-Sam68 was separated on Tris-Glycine gels and digested by trypsin: gel plugs were washed with B solution (200 mM TEAB, 50% acetonitrile) three times, each time incubating for 20 min, then washed with 100% acetonitrile and incubated for 10 min. After acetonitrile removal, gel plugs were air dried for 10 min, following by incubation in DTT solution (10 mM DTT, 50 mM TEAB) for 30 min at 60°C. Then gel plugs were incubated in iodocetamide solution (100 mM iodecetamide, 50 mM TEAB), followed by two washes with B solution, each time incubating for 20 min, then washed once with 100% acetonitrile and incubated for 10 min. After acetonitrile removal, gel plugs were air dried for 10 min and incubated in trypsin solution (50 ng trypsin, 50 mM TEAB) overnight at 37°C. After incubation, the supernatant was collected from the gel plugs into a new tube (A). After addition of 25% acetonitrile, 5% formic acid solution was added to gel plugs and samples were sonicated in a water bath for 10 min followed by collection of liquid into tube A. This step was repeated three times. Samples were concentrated using a speed vacuum centrifuge, resuspended in 2% acetonitrile, 0.1% formic acid. Reversed phase chromatography was used to separate tryptic peptides prior to MS analysis. Two columns were utilized, an Acclaim PepMap μ-pre-column cartridge 300 μm i.d. × 5 mm 5 μm 100 Å and an Acclaim PepMap RSLC 75 μm × 50 cm 2 μm 100 Å (Thermo Scientific). The columns were installed on an Ultimate 3000 RSLCnano system (Dionex). Mobile phase buffer A was composed of 0.1% formic acid in water and mobile phase B of 0.1% formic acid in acetonitrile. Samples were loaded onto the μ-pre-column equilibrated in 2% aqueous acetonitrile containing 0.1% trifluoroacetic acid for 5 min at 10 μl/min, after which peptides were eluted onto the analytical column at 250 nl/min by increasing the mobile phase B concentration from 4% B to 25% over 37 min, then to 35% B over 10 min, and to 90% B over 3 min, followed by a 10 min re-equilibration at 4% B. Eluting peptides were converted to gas-phase ions by means of electrospray ionization and analysed on a Thermo Orbitrap Fusion (Q-OT-qIT, Thermo Scientific). Survey scans of peptide precursors from 375 to 1575 *m/z* were performed at 120 K resolution (at 200 *m/z*) with a 2 × 10^5^ ion count target. Tandem MS was performed by isolation at 1.2 Th using the quadrupole, HCD fragmentation with normalized collision energy of 33 and rapid scan MS analysis in the ion trap. The MS^2^ ion count target was set to 5 × 10^3^ and the maximum injection time was 200 ms. Precursors with charge state 2–6 were selected and sampled for MS^2^. The dynamic exclusion duration was set to 25 s with a 10 ppm tolerance around the selected precursor and its isotopes. Monoisotopic precursor selection was turned on. The instrument was run in top speed mode with 2 s cycles. Data were searched using Mascot (version 2.6.1, Matrix Science Ltd, UK) against the *Homo sapiens* reference proteome database (www.uniprot.org/proteomes), and results were imported into Scaffold (version 5.1.0, Proteome Software Inc.). Spectra of modified peptides with Mascot delta score >0 were manually inspected to confirm sites of modification.

### Nuclear magnetic resonance

NMR experiments were recorded on a Bruker 600 MHz spectrometer equipped with triple-resonance ^1^H/^13^C/^15^N cryogenic probe. NMR measurements were performed in 50 M sodium phosphate buffer, pH 7.0, 150 mM NaCl and 10% D_2_O. All NMR spectra were processed using TopSpin software and analysed by CcpNmr Analysis (32).

Sam68 N- and C-terminal region backbone assignments were done using the following triple resonance experiments: HNCO, CBCA(CO)NH and HNCACB. In order to obtain an optimal signal with fewer peak overlaps, experiments were measured at 4°C.

(^1^H-^15^N)-HSQC spectra were recorded to probe the interaction between ^15^N-labelled N- or C-terminal regions and G8.5 or poly(C) unlabelled RNAs. The weighted chemical shift changes, Δδ(^1^H,^15^N), were calculated using the following equation: Δδ(H,N) = (Δδ^2^_H_ + Δδ^2^_N_ × 0.159)^1/2^.

Dissociation constants were obtained by fitting the chemical shift perturbation (CSP) data to the following equation:

$$\begin{equation*}{\rm{\Delta \delta }}obs = \Delta \delta max\left( {\frac{{a + b + Kd - \surd ( {{{( {a + b + Kd} )}}^2 - 4ab} ))}}{{2a}}} \right)\end{equation*}$$

where Δδobs is the average weight of the chemical shifts in the free and bound states and Δδmax is the maximal signal change upon saturation. *K*_d_ is the dissociation constant, and a and b are the total RNA and Sam68 C- or N-terminal region concentrations, respectively. Dissociation constants were calculated based on CSPs of eight distinct backbone resonances or based on the arginine side chain resonances.

### Phosphorylation of Sam68 C- and N-terminal regions

Phosphorylation of the Sam68 N- and C-termini was obtained by incubation with Cdk1/cyclin B (Merck, catalogue 14–450) at a Cdk1:Sam68 ratio of 5:1000 at 20°C, in the presence of 2 mM ATP, 5 mM MgCl_2_ and protease inhibitors in 50 mM sodium phosphate buffer pH 7.0 and 150 mM NaCl. First an initial (^1^H-^15^N)-HSQC was recorded at 4°C. Phosphorylation was then quantitatively monitored with consecutive 2D (^1^H-^15^N)-SOFAST-HMQC NMR experiments (33) for 16 h at 20°C, and a final (^1^H-^15^N)-HSQC at 4°C. The time course of the normalized amplitude of phosphorylated T33 and T317 is sigmoidal rather than hyperbolic and was fitted using a Hill function.

### Western blot of Sam68

HCT116 cells expressing the GFP-tagged wild-type (WT) or mutant Sam68 proteins were lysed in an equal packed cell volume of H200 buffer [20 mM HEPES pH 7.5, 200 mM NaCl, 1.5 mM MgCl_2_, 0.2 mM EDTA, 0.01% NP-40, 1 mM DTT and 1× protease inhibitor cocktail (Roche)]. The cell lysates were resolved on a 4–12% pre-cast sodium dodecylsulphate–polyacrylamide gel electrophoresis (SDS–PAGE) gel (Invitrogen) and transferred onto nitrocellulose membranes. Membranes were then probed with anti-Sam68 (Sigma-Aldrich, #07–415-I) and anti-γ-tubulin (Sigma-Aldrich, #T5326) primary antibodies and secondary IRDye-conjugated antibodies (800CW-Mouse and 680RD-Rabbit, LICOR, #926–32211 and #926–68071 respectively) before visualization using the LICOR Odyssey imaging system. Image processing and quantification were carried out using FIJI.

### RNA pulldown

HCT116 cells expressing the GFP-tagged WT or mutant Sam68 proteins were lysed in an equal packed cell volume of H200 buffer [20 mM HEPES pH 7.5, 200 mM NaCl, 1.5 mM MgCl_2_, 0.2 mM EDTA, 0.01% NP-40, 1 mM DTT and 1× protease inhibitor cocktail (Roche)]. Then 1 U/μl of RNAseIN ribonuclease inhibitor (Promega) and 1 μg of biotinylated G8.5 RNA were added to 250 μg of HCT116 whole-cell lysate, and the samples were incubated at room temperature for 30 min. Following this, 30 μl of Neutravidin beads (ThermoFisher Scientific) were added followed by a further incubation for 30 min at room temperature with resuspension every 5 min. The samples were centrifuged at 2500 *g* for 5 min and the supernatant was removed. The beads were then washed three times in 1 ml of H200 buffer. After the final wash, the beads were resuspended in an equal volume of H200 buffer, and 0.1 μg/μl of RNase A/T1 was added and incubated at 30°C for 15 min at 1400 rpm. Samples were centrifuged at 2500 *g* for 5 min and the supernatant containing biotinylated G8.5-bound protein was probed by western blot.

### Fluorescence microscopy

HCT116 cells were seeded on 1.5 mm coverslips (VWR) 24 h prior to transfection with pLEICS25 WTe or mutant GFP–Sam68. Transfected cells were cultured for 48 h, washed with PBS and fixed with 4% paraformaldehyde for 10 min. Paraformaldehyde was removed by three washes with PBS (Life Technologies), then DNA was stained with 300 nM 4′,6-diamidino-2-phenylindole (DAPI; ThermoFisher Scientific) for 5 min. Excess DAPI was removed by washing cells three times with PBS. Coverslips were mounted on slides using 3% (w/v) *n*-propyl gallate (Sigma) in an 80% glycerol solution and sealed with clear nail varnish. Cells were imaged using a Zeiss LSM 980 Airyscan 2 microscope in confocal mode with a Plan-Apochromat ×63/1.40 Oil DIC f/ELYRA lens. A 405 nm laser was used to excite DAPI and was detected within 415–475 nm; a 488 nm laser was used to excite GFP and was detected within 495–555 nm. Sam68 localization patterns were assigned and counted manually for 50–100 cells per sample. Statistical analysis was done using GraphPad/Prism. The comparison of means between independent groups was performed using unpaired *t*-test.

### Splicing assays

The splicing activities of the Sam68 WT and mutant proteins were assessed in HCT116 cells using a minigene assay as previously described (34). In brief, HCT116 cells were transfected with WT or mutant GFP–Sam68 and CD44 or Bcl-x minigenes. Transfected cells were harvested after 48 h. RNA was isolated and purified using the Monarch Total RNA Miniprep Kit (Biolabs) following the manufacturer's protocol. cDNA was transcribed using the High-Capacity cDNA Reverse Transcription Kit (ThermoFisher Scientific). The Bcl-x minigene was amplified with the forward primer 5′-AGTTTGAACTGCGGTACCGGCG-3′ and reverse primer 5′-TCATTTGTATTTCCAAGGAGTTAACCTC-3′. The CD44 minigene was amplified with the forward primer 5′-CTGTCCTCTGCAAATGCTGTTTATGAAC-3′ and reverse primer 5′-AATAACCAGCACGTTGCCCAGGAG-3′. PCR products were separated on a 1.5% agarose gel and visualized with a U:GENIUS gel imaging system (Syngene). Band intensities were quantified with ImageJ and statistical analysis was done using GraphPad/Prism. The comparison of means between independent groups was performed using unpaired *t*-test.

### Cell cycle progression assays

Cell cycle profiles were determined by measuring cellular DNA content by flow cytometry. At 48 h post-transfection, cells were collected by trypsinization, washed with PBS and fixed with ice-cold 70% ethanol at –20°C overnight. Cells were washed three times with PBS and resuspended in 300 μl of PBS, then RNase A (200 μg/ml final concentration) and propidium iodide (PI) (20 μg/ml final concentration) were added to the samples and incubated at 4°C overnight in the dark. Cell cycle profiles were determined using CytoFlex (Beckman) and analysed using FlowJo. Statistical analysis was done using GraphPad/Prism. The comparison of means between independent groups was performed using unpaired *t*-test.

### Apoptosis assays

Apoptosis was determined using the Annexin V-APC PI kit (Biolegend). At 48 h post-transfection, cells were collected by trypsinization and washed with PBS. The cells were resuspended in 300 μl of binding buffer, then 5 μl of Annexin V-APC (stock concentration of 0.5 mg/ml) and 5 μl of PI (stock concentration of 4 mg/ml) were added to the samples for 10 min and incubated in the dark. Apoptosis were determined using CytoFlex and analysed using FlowJo. Statistical analysis was done using GraphPad/Prism. The comparison of means between independent groups was performed using unpaired *t*-test.

### Proliferation assays

At 24 h before transfection, HCT116 cells were seeded as 2500 cells per well in 96-well plates. Cells in each well were transfected with 0.15 μg of plasmid DNA and the medium was replaced 24 h after transfection. Cell proliferation was tested at different time points by addition of 5 μl of CellTiter-GLo (Promega) to each well and followed by quantification of the presence of ATP using the Hidex Sense plate reader.

## RESULTS

### Mapping of Sam68 PTMs in HEK293 and HCT116 cells

**Figure 1.**
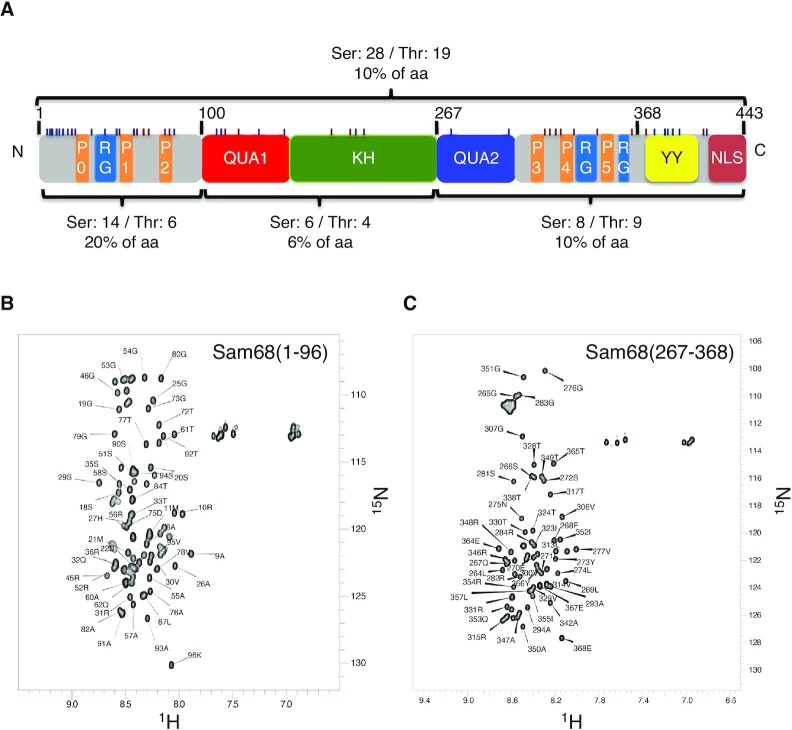
Domain organization of Sam68 and NMR analysis of the N- and C-terminal regions. (**A**) Sam68 is a 443 amino acid protein. The QUA1 and KH domains are responsible for dimerization and RNA binding. The N-terminal region (residues 1–96), the QUA2 region (267–283) and the C-terminal region (residues 284–443) are predicted to be intrinsically disordered and contain regulatory motifs such as proline-rich motifs (P0–P5), RG-rich motifs (RG), a tyrosine-rich region (YY) and a nuclear localization signal (NLS). The number and percentage of serines and threonines for each region/domain are indicated and their position is indicated by small bars (blue for Ser and purple for Thr). (**B, C**) NMR (^1^H-^15^N)-HSQC spectra of the Sam68 N-terminus (residues 1–96) (B) and C-terminus (residues 267–368) at 4°C (C). The assignment of the backbone amide resonances is indicated.

The amino acid sequence of Sam68 contains 28 serines and 19 threonines that are mainly located in the N- and C-terminal regulatory regions (Figure 1A). To map serines and threonines phosphorylated in cells, Flag-tagged full-length human Sam68 was transfected in HEK293 and HCT116 cells. At 48 h post-transfection, asynchronous cells were harvested and Flag-Sam68 was purified using a flag antibody, and digested with trypsin. PTMs were identified by liquid chromatography–tandem MS (LC-MS/MS; Table 1). The datasets did not provide any sequence coverage of the tyrosine-rich region of Sam68, and no phosphotyrosine residues were detected. This was expected, as digestion with trypsin gives very large peptides for this region. However, as expected, we observed methylation of multiple arginines and acetylation of multiple lysines as previously reported (23,25). In terms of serine and threonine (S/T) phosphorylation, we observed phosphorylation of either S18 or S20 (S18/S20) and of either T33 or S35 (T33/S35) in HEK293T cells; and of either S18 or S20 and of either T33 or S35 and S113 in HCT116 cells (Table 1).

**Table 1.** List of Sam68 post-translational modifications identified by LC-MS/MS

	HCT116 unsynchronized	Hek293 unsynchronized	HEK293 S phase	HEK293 M phase	HEK293 G_1_ phase
Arginine methylation	R43, R45, R52, R282, R284, R289, R291, R302, R304, R310, R315, R320, R325, R331, R340	R36, R186, R282, R284, R289, R291, R302, R304, R310, R325, R331, R340	R4, R43, R45, R52, R186, R213, R282, R284, R289, R291, R302, R304, R310, R320, R325, R331, R340, R345	R43, R45, R52, R186, R282, R284, R289, R291, R302, R304, R310, R315, R320, R325, R331, R340, R345	R282, R284, R289, R291, R302, R325, R331
Lysine acetylation	K111, K139	K139, K194, K206	K131, K139, K208	K111, K131, K138, K139, K152, K206, K208, K432	K111, K131, K139, K169, K175, K194, K206,
Serine/threonine phosphorylation	S18/S20 ^a^ , S35/T33^b^, S113	S18/S20^a^, S35/T33^b^	S20	S18/S20^a^, T33, S150, T317	S20, S58, S70

^a^We observed single phosphorylation of the peptide containing S18 and S20, indicating that we cannot differentiate which serine is phosphorylated (HCT116), or that two populations co-exist in the same sample one with S18 phosphorylated, another with S20 phosphorylated (HEK293 unsynchronized and M phase).

^b^We observed single phosphorylation of the peptide containing S35 and T33, indicating that we cannot differentiate which amino acid is phosphorylated (HCT116), or that two populations co-exist in the same sample one with S35 phosphorylated, another with T33 phosphorylated (HEK293).

Next, we investigated the PTMs of Sam68 in HEK293 cells at different stages of the cell cycle by treating cells with either nocodazole (G_1_ and M phases) or hydroxyurea (S phase). The enrichment in specific phases of the cell cycle was verified by flow cytometry (Supplementary Figure S1) and samples were analysed by LC-MS/MS. S18/S20 phosphorylation was observed in all stages of the cell cycle. Additionally, S58 and S70 were phosphorylated during G_1_. These two specific phosphorylation events could be mediated by Erk1 as previously suggested (7). Interestingly, we identified T33 and T317 phosphorylation in mitosis. These phosphorylation events could be mediated by Cdk1/cyclin B (21).

### Cdk1 phosphorylates Sam68 at T33 and T317 *in vitro*

**Figure 2.**
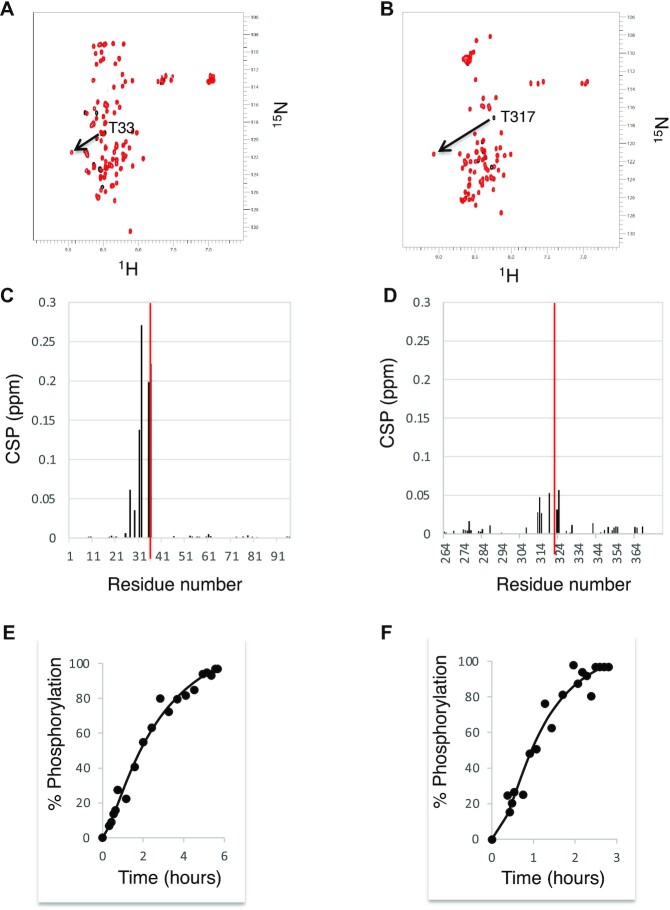
The Sam68 N- and C-termini are phosphorylated at T33 and T317 by Cdk1. (**A, B**) (^1^H-^15^N)-HSQC spectra overlay of the Sam68 N-terminus (A) and C-terminus (B) before (black) and after 16 h incubation with commercial active Cdk-1/cyclin B (red) at 4°C. T33 and T317 resonance peaks are indicated. (**C, D**) CSP of backbone amides as a function of the N-terminal (C) and C-terminal (D) amino acid sequence upon Cdk1 phosphorylation. For clarity, the CSPs of T33 and T317 are represented as red bars and are not to scale. (**E, F**) Normalized intensity of the phosphorylated T33 (E) and T317 (F) backbone amide peak as a function of time after Cdk1/cyclin B addition. The data were fitted using a Hill function.

To precisely investigate the phosphorylation of Sam68 by Cdk1 *in vitro*, we used NMR spectroscopy that is very powerful for the study of PTMs at atomic resolution (35). The N-terminal (residues 1–96) and C-terminal (residues 267–368) regions of Sam68 were expressed in *E. coli* in the presence of [^15^N]ammonium chloride and [^13^C]glucose before purification. The NMR spectra show that these regions are intrinsically disordered and 72% and 65% of the non-proline residue backbone resonances for the N- and C-terminal regions, respectively, could be unambiguously assigned (Figure 1B, C). Next, these purified regions were incubated with ATP, MgCl_2_ and active Cdk1/cyclin B. Phosphorylation was quantitatively monitored by recording a (^1^H-^15^N)-HSQC at time zero at 4°C, followed by consecutive 2D (^1^H-^15^N)-SOFAST-HMQC NMR experiments for 16 h at 20°C, and a final (^1^H-^15^N)-HSQC at 4°C. During the experiment, the resonance peaks of T33 and T317 shifted downfield in both ^1^H and ^15^N dimensions, a typical signature of serine or threonine phosphorylation (36) (Figure 2A, B). Resonances corresponding to other threonine and serine residues were not affected by the addition of Cdk1/cyclin B, indicating that only T33 and T317 were phosphorylated by Cdk1 *in vitro*. Incubation of the N-terminus with Cdk1 induced CSPs of residues surrounding T33 (notably H27, S29, R31, Q32, R35 and Q36) (Figure 2C), suggesting a possible conformational change upon T33 phosphorylation. In contrast, phosphorylation of T317 in the C-terminal region only induced minor chemical shift changes to the surrounding residues, suggesting no major conformational changes (Figure 2D).

Analysis of the (^1^H-^15^N)-SOFAST-HMQC spectra at different times after addition of Cdk1 allowed us to investigate the kinetics of T33 and T317 phosphorylation by Cdk1 (Figure 2E, F). Interestingly, phosphorylation of these residues did not follow a Michaelian response but rather a sigmoidal ultrasensitive response. Such a response is not uncommon in cell signalling systems (37,38) and has already been suggested for other Cdk1 substrates (39–41). Overall, the complete phosphorylation of T317 is faster (2.5 h) than that of T33 (5 h). This is consistent with the amino acid sequence surrounding the phosphorylated residues, T_33_PSR and T_317_PVR. The target sequence for Cdk1 is (S/T)PX(K/R), where X can be any amino acid. However, preferential amino acids have been described at this position, with a valine predicted to provide a better Cdk1 target than a serine (42).

### The N- and C-terminal regions of Sam68 bind RNA

Like other STAR proteins, Sam68 binds predominantly and specifically to RNA through its central STAR domain (18,43–45). However, the dissociation constant of full-length Sam68 for SELEX-derived RNAs was reported to be in the low nanomolar range (46,47), while we found that the isolated STAR domain binds the same RNA sequences with dissociation constants in the low micromolar range (18). This suggests that regions of Sam68 outside the STAR domain could contribute to RNA binding. Sam68 N- and C-terminal regions contain multiple RG-rich regions, and it is well established that RG-rich regions of many proteins have RNA binding properties (48). Accordingly, it has been shown that Sam68’s RG-rich regions are capable of binding RNA non-specifically (49).

**Figure 3.**
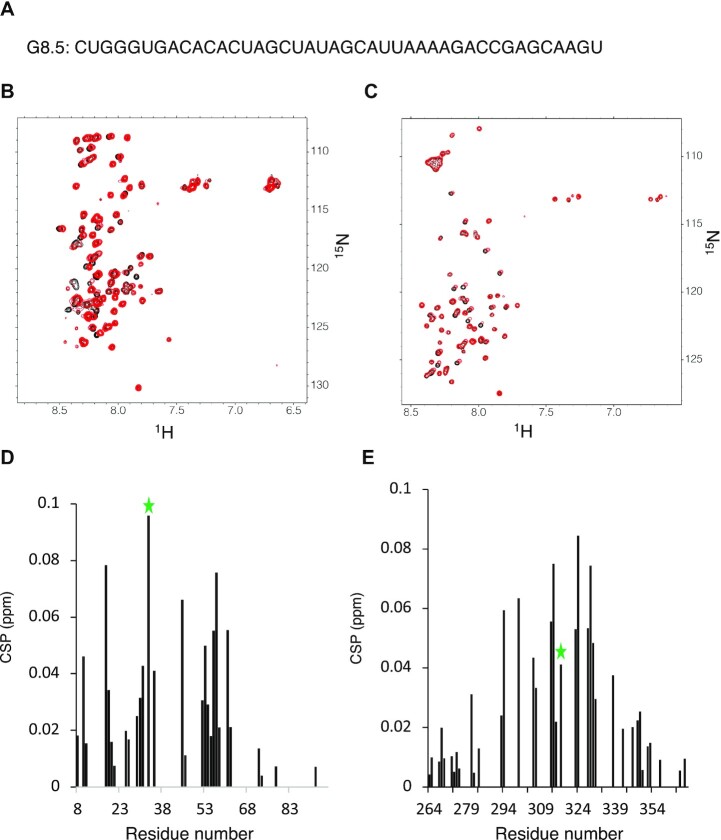
The N- and C-terminal regions of Sam68 bind RNA. (**A**) Nucleotide sequence of the G8.5 RNA identified previously as a high-affinity binder of Sam68 (46). (**B, C**) (^1^H-^15^N)-HSQC spectra of the Sam68 N-terminus (B) and C-terminus (C) before (black) and after (red) addition of excess G8.5 RNA (protein:RNA molar ratio of 1:2) at 4°C. (**D, E**) Chemical shift perturbation of the Sam68 N- (D) and C-terminal (E) backbone resonances upon RNA interaction as a function of the amino acid sequence. T33 and T317 CSPs are labelled with a green star. CSPs >0.025 (average CSP) are considered significant

We therefore used NMR spectroscopy to investigate the binding of purified Sam68 N- and C-terminal regions with the RNA sequence G8.5 that was previously identified as a high affinity Sam68 binder by SELEX (46) (Figure 3A). Increasing the G8.5 molar ratio with the Sam68 N- and C-termini induced chemical shift perturbations, consistent with the formation of a complex between these regions and the RNA (Figure 3B, C). These results demonstrate that the Sam68 N- and C-terminal regions can bind RNA independently of the rest of the protein. Analysis of the titration data suggests that both domains bind the G.8 RNA with dissociation constants in the low micromolar range (1–10 μM for the N-terminus and 30–70 μM for the C-terminus) (Supplementary Figure S2), very similar to the affinity of the STAR domain for the same RNA sequence (18).

Analysis of the chemical shift mapping shows that RNA binding affects most residues of the N- and C-termini, in particular residues 10–61 for the N-terminus and 281–338 for the C-terminus (Figure 3D, E). This is consistent with the presence of multiple RG-rich motifs in the sequence. However, it could also indicate that RNA binding induces conformational changes to the Sam68 N- and C-termini. To investigate whether the RNA binding of the Sam68 N- and C-termini is specific to the G8.5 RNA, we performed a similar chemical shift mapping using a poly(C) RNA of the same length (Supplementary Figure S3). In that case, the addition of the RNA induces only minor chemical shift perturbation, suggesting that the N- and C-terminal regions of Sam68 bind specifically to the G8.5 RNA. Interestingly, addition of the G8.5 RNA induces significant perturbation of the chemical shifts of T33 and T317, demonstrating that their chemical environment is affected either by direct binding to the RNA or by RNA-induced conformational changes. This implies that phosphorylation of these residues could modulate RNA binding through the N- and C-terminal regions.

### Phosphorylation of Sam68 N- and C-terminal regions by Cdk1 decreases their RNA binding ability *in vitro*

**Figure 4.**
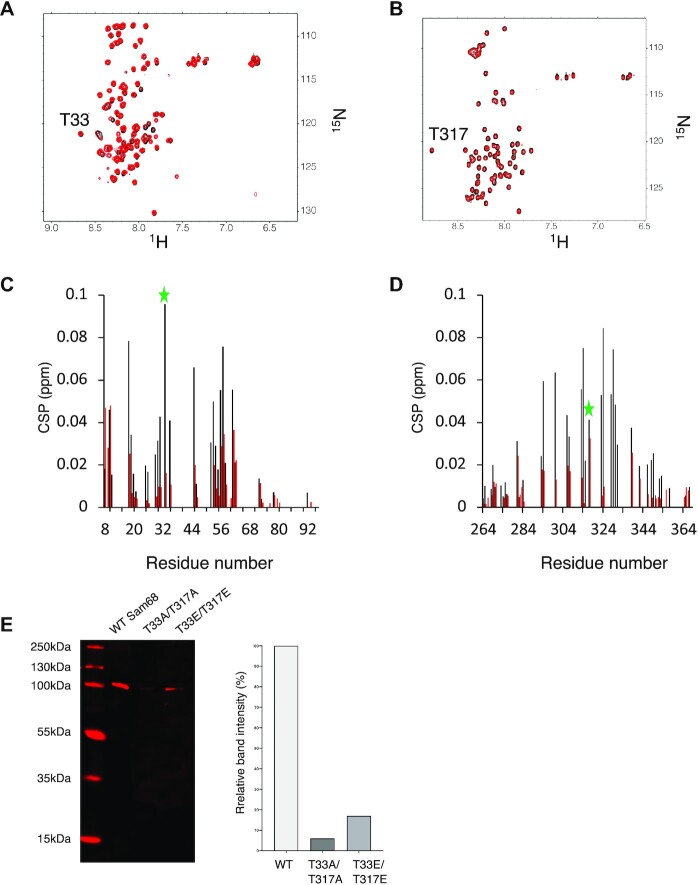
T33 and T317 phosphorylation reduces the RNA binding ability of the Sam68 N- and C-termini. (**A, B**) (^1^H-^15^N)-HSQC spectra of the Cdk1-phosphorylated Sam68 N-terminus (A) and C-terminus (B) before (black) and after (red) addition of 2 molar equivalent of G8.5 RNA at 4°C. Resonances of T33 and T317 are indicated. (**C, D**) CSP of the Sam68 N-terminus (C) and C-terminus (D) in their unphosphorylated (black) or Cdk-1 phosphorylated (red) forms upon RNA binding. T33 and T317 CSPs are labelled with a green star. CSPs >0.025 (average CSP) are considered significant. (**E**) RNA pulldown of GFP–Sam68 WT or mutant by biotinylated G8.5 RNA.

We next investigated whether the phosphorylation of T33 and T317 by Cdk1 had an effect on the RNA binding ability of Sam68 N- and C-termini. For this purpose, we measured NMR (^1^H-^15^N)-HSQC spectra of Cdk1-phosphorylated Sam68 N- and C-terminal regions in the absence or the presence of an excess of G8.5 RNA (Figure 4A, B). The phosphorylated form of the Sam68 N-terminus was still able to bind the G8.5 RNA but the intensity of the CSPs was strongly reduced compared with the non-phosphorylated regions (Figure 4C). This indicates that phosphorylation of T33 reduces but does not abolish its RNA binding ability. Analysis of the NMR titration experiment suggests that the *K*_d_ of the phosphorylated Sam68 N-terminus for the G8.5 RNA is ∼100 μM (Supplementary Figure S4), significantly higher than the *K*_d_ with the unphosphorylated form. For the phosphorylated C-terminus, the chemical shift changes upon RNA addition were too small (Figure 4D) to allow us to derive a meaningful estimation of the *K*_d_, suggesting that the dissociation constant of the T317-phosphoryled C-terminus to the RNA is >100 μM. Together, our data demonstrate that the N- and C-terminal regions of Sam68 are capable of binding RNA and that phosphorylation of T33 and T317 by Cdk1 significantly reduces their RNA binding ability.

### Phospho-null and phospho-mimetic mutants of Sam68 have reduced RNA binding ability in HCT116 cells

To investigate the consequences of Sam68 T33 and T317 phosphorylation on RNA binding, we created a phospho-null (T33A/T317A) and a phospho-mimetic (T33E/T317E) GFP-tagged Sam68 mutant. Following transfection in HCT116 cells, we ensured that the transfection efficiency was at least 70% and that the levels of expression of the WT and mutants were similar (Supplementary Figure S5). Next, we incubated the cell lysates with biotinylated G8.5 RNA and analysed the binding by a pulldown experiment and western blot. As expected, WT Sam68 binds the G8.5 RNA (Figure 4E). However, the ability of both T33A/T317A and T33E/T317E mutants to bind the RNA is much lower (Figure 4E). Importantly, the reduced affinity of the T33E/T317E mutant is consistent with our *in vitro* NMR data and confirms that phosphorylation of Sam68 T33 and T317 affects its RNA binding ability in a cellular context. The reduction in RNA binding affinity observed for the T33A/T317A mutant also suggests that the two threonines T33 and T317 are involved and crucial for RNA binding and that any modification of these amino acids, through either phosphorylation or mutation, affect the functions of Sam68. This would be consistent with the fact that following transfection of Sam68 mutants, the endogenous Sam68 protein can be detected by western blot (Supplementary Figure S5B). Indeed, it has previously been demonstrated that Sam68 expression is regulated by an autoinhibitory feedback loop (50). The inability of the mutants to bind RNA would abolish this feedback loop, leading to increased levels of endogenous Sam68 following transfection with the mutants compared with the WT.

### T33 and T317 mutations alter the cellular localization of Sam68

Sam68 is diffusely distributed throughout the nucleus in HEK293, mouse embryo fibroblast (MEF), HF-7650 and REF-52 cells, while in some cell lines such as HeLa, BT-20 and Hs578T, Sam68 is also localized in nuclear speckles, called Sam68 nuclear bodies (SNBs) (51). Mutations and deletions of some regions of Sam68 (notably RNA-binding mutants in the STAR domain) have been shown to result in the relocalization of Sam68 to different compartments, leading to the description of seven localization patterns for WT and mutant Sam68 ranging from diffuse in the nucleus with some SNBs to only one SNB or even fibrous cytoplasmic structures (51). Notably, all mutations in the KH RNA-binding domain of Sam68 led to a significant decrease in the number of cells displaying a diffuse nuclear localization and an increase in the number of cells displaying nuclear and/or cytoplasmic speckles, indicating that any interference with the RNA binding properties of Sam68 leads to increased SNB formation and/or cytoplasmic localization (51).

**Figure 5.**
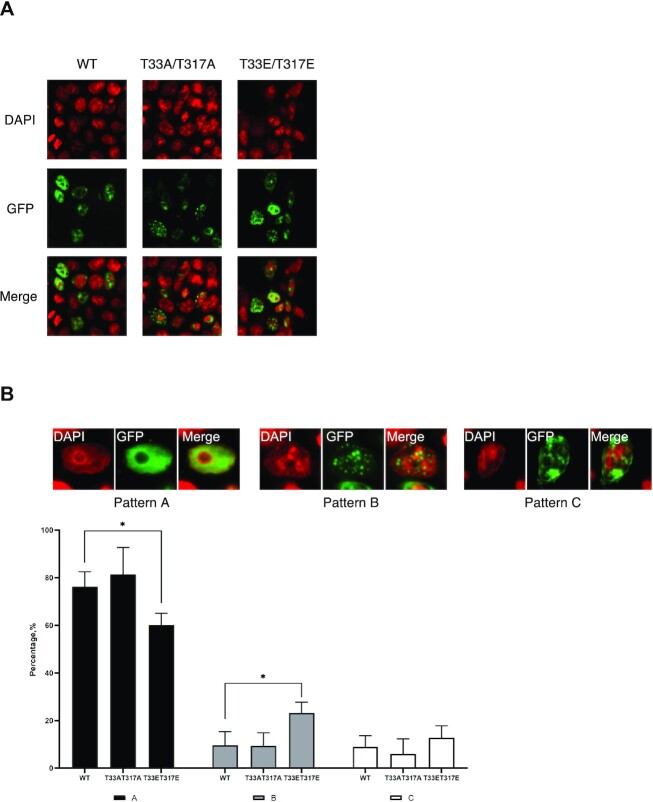
Cellular localization of Sam68 WT, T33A/T317A and T33E/T317E mutants in HCT116 cells. (**A**) Confocal fluorescence images of HCT116 cells transfected with either GFP-tagged Sam68 WT or mutants. DAPI is shown in red and GFP in green. (**B**) Representative images of three different classes (patterns A–C) of cellular localization of Sam68 WT and mutants in HCT116 cells (top) and percentage of cells displaying Sam68 or mutants localized in each class. Error bars represent the standard deviation of three independent transfection experiments of WT and mutant Sam68. The localization class was assessed using 50–100 GFP-positive cells in each independent experiment (bottom). *P*-values were calculated using an independent two-sample *t*-test (*P <0.05).

To investigate the consequences of Sam68 T33 and T317 phosphorylation on cellular localization, we compared the localization of GFP–Sam68 WT, T33A/T317A and T33E/T317E mutants in HCT116 cells (Figure 5A). As expected, WT Sam68 is mainly diffused in the nucleus with sometimes few SNBs (76.2 ± 6.3%, pattern A), with a small proportion of cells displaying Sam68 exclusively in SNBs (9.7 ± 5.7%, pattern B) or localized in both the nucleus and the cytoplasm (9.0 ± 4.6%, pattern C) (Figure 5B). In contrast, the T33E/T317E mutant displayed significant localization differences when compared with WT Sam68, with a significant decrease in the percentage of cells displaying pattern A (60.1 ± 4.9) and a 2-fold increase in the percentage of cells displaying pattern B (23.2 ± 4.5) (Figure 5B). These changes in localization are similar to previously reported mutations in the KH RNA-binding domain of Sam68 (51).

### T33 and T317 mutants have reduced Sam68 splicing activity

**Figure 6.**
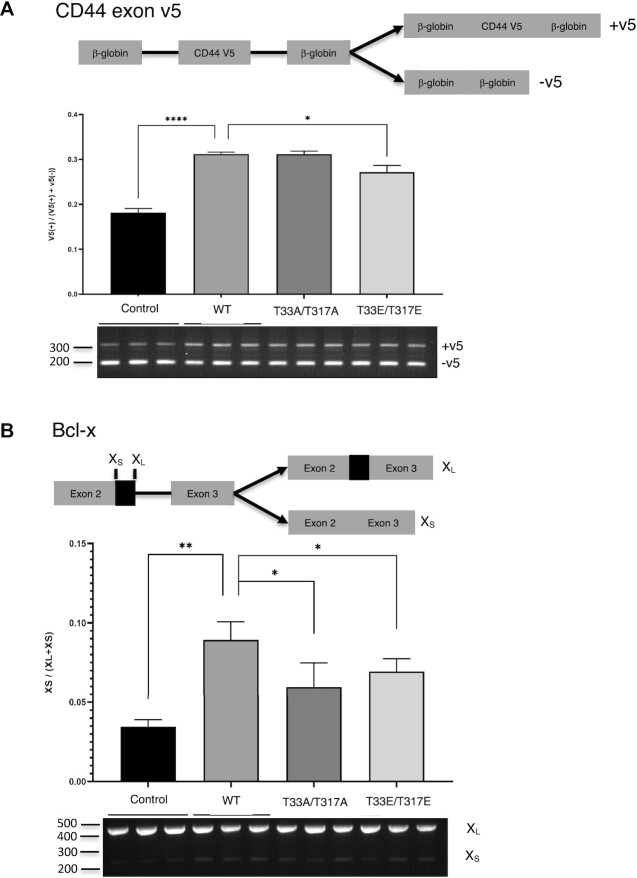
Splicing activity of Sam68 WT and phospho-mimetic mutants on CD44 exon v5 and Bcl-x minigenes in HCT116 cells. Effect of Sam68 WT, T33E, T317E and T33E/T317E transfection on the alternative splicing of CD44 exon v5 (**A**) and Bcl-x (**B**) minigenes. Bottom: agarose gel electrophoresis showing splicing of the minigenes in response to co-transfected proteins. Top: quantification of biological replicates from three independent co-transfection experiments. Bar chart plotting and analysis were performed using GraphPad Prism. Error bars represent the standard deviation of three independent experiments. *P*-values were calculated using an independent two-sample *t*-test (*P <0.1, **P <0.01, ****P <0.0001). Uncropped gels are shown in Supplementary Figure S9.

To test whether phosphorylation of Sam68 at T33 and T317 might affect its splicing activity, we investigated the ability of the T33A/T317A and T33E/T317E mutants to regulate splicing of CD44 and Bcl-x minigenes (7,8) in HCT116 cells (Figure 6). As previously reported, transfection of WT Sam68 induced the inclusion of exon v5 in CD44 (Figure 6A) and increased the Bcl-x_S_/Bcl-x_L_ ratio (Figure 6B). However, the T33E/T317E mutant was significantly less efficient at including CD44 exon v5 compared with the WT Sam68 (0.27 ± 0.01 for T33E/T317E compared with 0.31 ± 0.00 for WT Sam68), and both mutants were less efficient at shifting splicing towards the X_S_ isoform [X_S_/(X_L_ + X_S_) ratio of 0.06 ± 0.02 and 0.07 ± 0.01 for T33A/T317A and T33E/T317E compared with 0.09 ± 0.01 for WT Sam68]. Hence, phosphorylation of Sam68 T33 and T317 attenuates Sam68 regulatory activity on CD44 and Bcl-x splicing.

### T33 and T317 mutants affect cell cycle progression, decrease apoptosis and increase proliferation

**Figure 7.**
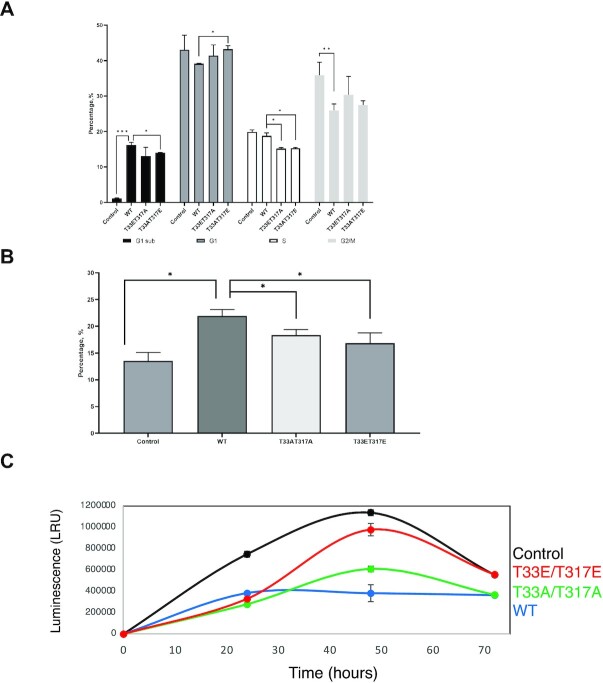
Effect of Sam68 WT or phospho-mimetic mutants on cell cycle progression, apoptosis and proliferation of HCT116 cells. (**A**) Percentage of cells in sub-G_1_ (black), G_1_/G_0_ (dark grey), S (white) and G_2_/M (light grey) phases 48 h after transfection with Sam68 WT or mutants (flow cytometry images are presented in Supplementary Figure S6). Controls are untransfected cells. (**B**) Percentage of apoptotic cells 48 h after transfection with Sam68 WT or mutants (flow cytometry images are presented in Supplementary Figure S7). (**C**) Normalized cell proliferation increase between 24 and 96 h after transfection with Sam68 WT or mutants. Controls are untransfected cells. Error bars represent the standard deviation of two independent experiments. *P*-values were calculated using an independent two-sample *t*-test (statistical significance shown as: *P <0.05, **P <0.005 and ***P <0.0005).

Sam68 is a key regulator of cell cycle progression and apoptosis (52). We therefore performed flow cytometry with DNA staining to investigate the consequences of transfecting Sam68 WT and mutants on HCT116 cell cycle progression (Supplementary Figure S6). Significant changes could be observed in the relative proportions of sub-G_1_, G_1_ and G_2_/M populations upon expression of either WT Sam68 or the mutants (Figure 7A). Expression of Sam68 WT significantly decreased (from 35.9 ± 3.7% to 26.0 ± 1.8%) the G_2_/M population and significantly increased (from 1.2 ± 0.1% to 16.2 ± 0.8%) the sub-G_1_ population compared with untransfected cells. In contrast, expression of Sam68 T33A/T317A and T33E/T317E mutants resulted in increased G_2_/M populations (30.4 ± 5.2% and 27.5 ± 1.2, respectively) compared with WT expression. Moreover, Sam68 T33E/T317E transfection resulted in a significant decrease in the sub-G_1_ population (14.0 ± 0.1%) and S population (15.3 ± 0.2), and an increase in the G_1_ population (43.2 ± 1.0%) compared with the WT.

We next investigated the role of Sam68 T33 and T317 phosphorylation in apoptosis regulation using the Annexin V-APC PI flow cytometry assay (Supplementary Figure S7). Compared with untransfected cells, transfection of Sam68 WT increased the percentage of cells undergoing apoptosis as expected (from 12.1 ± 1.6% to 23.0 ± 1.7%), but this increase was significantly smaller upon transfection of the mutants (18.8 ± 1.5 and 16.6 ± 2.0 for T33AT317A and T33E/T317E, respectively) (Figure 7B).

Next, we investigated the rate of proliferation of HCT116 cells upon transfection of WT Sam68 or mutants by CellTiter-Glo cell viability assay. Consistent with an increase of apoptosis when the Sam68 WT is transfected, the rate of proliferation is lower in Sam68 WT-transfected cells than in untransfected cells (Figure 7C), indicating that WT Sam68 decreases HCT116 proliferation. However, the rate of cell proliferation was significantly higher following transfection of the T33E/T317E phospho-mimetic mutant compared with transfection with the WT Sam68 (Figure 7C).

Altogether, our data demonstrate that phosphorylation of Sam68 at T33 and T317 alters its regulatory role in cell cycle progression and apoptosis, leading to increased proliferation of HCT116 cells.

## DISCUSSION

It is well established that regulation of alternative splicing by splicing factors is modulated by cell signalling pathways (2,3). Indeed the large majority of splicing factors possess IDRs that are target sites for PTMs, and these modifications affect the function of splicing factors through various mechanisms, such as changes in cellular localization or RNA binding affinity (53,54). However, in most cases, the molecular details of how PTMs affect splicing factor functions remain unclear. In the case of Sam68, it is known that phosphorylation, acetylation and methylation regulate its localization and function in alternative splicing [reviewed in (27)], but the effects of these modifications at the atomic level are largely unknown. In terms of Ser/Thr phosphorylation, as stated above, Sam68 has been shown to be a target for Cdk1 (21), Erk1 (7), Nek2 (22) and possibly CAMK IV (30). However, the precise mapping of the phosphorylation sites and the mechanisms of these modifications on Sam68 functions remain largely unknown. It has been suggested that CAMK IV phosphorylates Ser20 of Sam68 in neurons in a depolarization-dependent manner and that this phosphorylation event affects Sam68’s regulation of Neurexin 1 pre-mRNA (30). CamK IV expression is mainly restricted to the brain in normal conditions, but it is also expressed in various types of cancer cells [reviewed in (55)]. Interestingly, our MS data identified the phosphorylation of S18 and/or S20 in both HEK293 and HCT116 cells, and in all stages of the cell cycle. This suggests either that CAMK IV is expressed at sufficient levels in these cell lines or that another kinase, yet to be identified, is responsible for this phosphorylation event. S20 phosphorylation of Sam68 has been observed in many proteomic studies (https://www.phosphosite.org/proteinAction.action?id=3642). Sam68 is also a target of Erk1 (7), but the precise identification of which Ser or Thr is phosphorylated by Erk1 remains unknown. Mutation of three Ser/Thr in the N-terminal region of murine Sam68 (S58, T71 and T84) by Ala (phospho-null mutants) reduced the Sam68-induced inclusion of CD44 exon v5, suggesting that at least one of these amino acids is phosphorylated by Erk1, and Erk-1 mediated phosphorylation of Sam68 enhances its splicing regulation activity. We did observe S58 phosphorylation but not T84 (T71 is not present in human Sam68) and only in cells arrested in G_1_ phase in our MS data. Since we show that phospho-null mutants of Sam68 T33A/T317A have similar effects as the phospho-mimetic mutants T33E/T317E in terms of RNA binding and splicing regulation, it is possible that S58, T71 and/or T84 are directly involved in RNA binding and therefore the reported effects of these mutations on Sam68 splicing function might not be due to a lack of phosphorylation. Accordingly, another study demonstrated that phosphorylation of Sam68 by Erk1 reduces its binding affinity for the CD44 pre-mRNA (29). Finally Sam68 is also phosphorylated by Nek2, and this phosphorylation event stimulates Sam68 splicing activity on CD44 exon v5 (22). Sam68 truncation showed that both the N- and C-terminal regions of Sam68 are phosphorylated *in vitro* by Nek2, but the precise identification of Nek2-phosphorylated residues was not investigated. Here we have investigated the phosphorylation of Sam68 by Cdk1/cyclin B at the atomic level and demonstrated that Cdk1 phosphorylates the T33 and T317 residues located in the N- and C-terminal IDRs of Sam68, respectively. We further show that phosphorylation of these residues reduces the binding of these regions to RNA and, most probaby as a consequence, alters Sam68 localization and reduces its splicing activity.

How the phosphorylation of Sam68 T33 and T317 reduces the RNA binding affinity of the N- and C-terminal regions of Sam68 remains to be elucidated. One possibility is that T33 and T317 are directly involved in RNA binding and that the addition of a negatively charged phosphate group induces an electrostatic repulsion with the negatively charged RNA. This would be consistent with our data showing that any modification of Sam68 T33 and T317, which can be either phosphorylation or mutation to alanine or glutamate, strongly reduces the RNA binding ability of Sam68 (Figure 4). Another possibility is that Sam68 N- and C-terminal regions bind RNA through their arginine side chains and, upon phosphorylation, the phosphate group forms a salt bridge with nearby arginine residues, preventing them from binding to RNA. Indeed it has been shown that phosphorylated amino acids can form very stable salt bridges with two arginine guanidino groups (56). This would be consistent with our NMR data showing that both RNA and phosphorylation induce chemical shift changes of the arginine side chain Nϵ (Supplementary Figure S8) and that both T33 and T317 are surrounded by nearby arginines, R31 and R36 in the N-terminal region and R315 and R320 in the C-terminal region.

**Figure 8.**
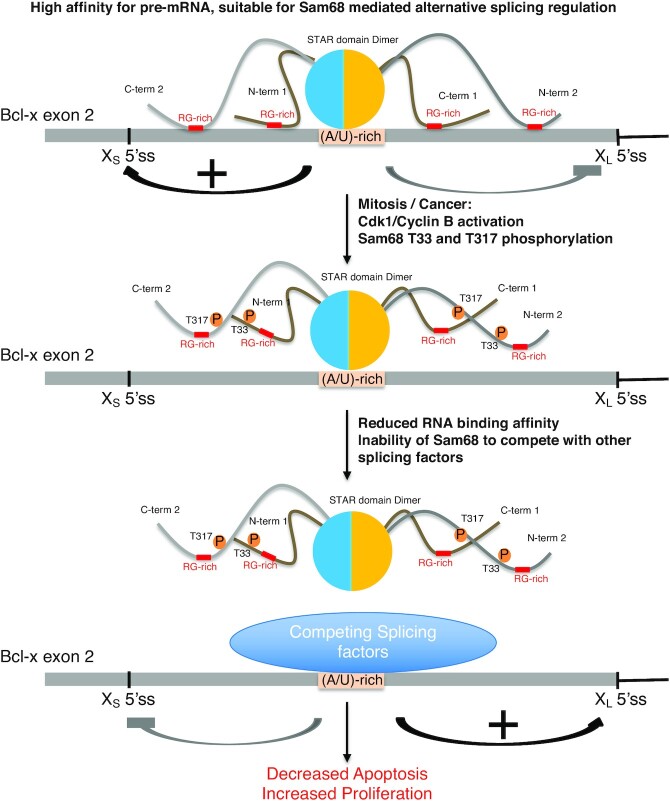
Model of splicing regulation by Cdk-1 mediated phosphorylation of Sam68/KHDRBS1. In interphase, Cdk-1 is inactive, so T33 and T317 of Sam68 are unphosphorylated. Sam68 would therefore bind (AU)-rich regions of the pre-mRNA specifically through its STAR homodimerization domain with a dissociation constant (*K*_d_) in the low micromolar range, and the N- and C-terminal regions would anchor the protein to the RNA, increasing the affinity of full-length Sam68 for RNA (*K*_d_ in the low nanomolar range). During mitosis or in cancer cells, activation of Cdk-1/cyclin B induces the phosphorylation of T33 and T317, leading to the dissociation of the N- and C-terminal anchoring region from the RNA and, therefore, a weakening in RNA binding by full-length Sam68, making Sam68 less effective at competing with other splicing factors for RNA binding. As a consequence, Sam68 activity in splicing regulation is reduced, leading to a decrease in cell apoptosis and an increase in proliferation.

Together, our data suggest a mechanistic model for the role of Cdk1 phosphorylation in Sam68 functions (Figure 8). In normal interphase cells, T33 and T317 of Sam68 are unphosphorylated, allowing its N- and C-terminal regions to bind RNA, most probably through their RG-rich regions and possibly directly by these two threonines. In this state, the STAR domain of Sam68 binds its pre-mRNA substrate specifically by recognizing the sequence (A/U)AA-N>15-(A/U)AA with a weak to medium affinity (dissociation constant in the micromolar range) (18). The primary binding of the STAR domain to the pre-mRNA brings Sam68 N- and C-terminal IDRs in close proximity to the RNA. As the STAR domain also promotes dimerization, two N-terminal and two C-terminal IDRs can bind the RNA, anchoring the protein to its target pre-mRNA (Figure 8, top), leading to high affinity RNA binding [dissociation constant in the low nanomolar range as reported previously for full-length Sam68 (46,47)]. Upon mitotic entry—or potentially in certain cancers—Cdk1 activity increases, leading to Sam68 phosphorylation at T33 and T317. These phosphorylation events reduce the RNA binding affinity and induce the release of Sam68 N- and C-terminal IDRs from the pre-mRNA (Figure 8, middle). The weakened affinity of phosphorylated Sam68 for the RNA reduces its ability to compete with other splicing factors and leads to its release from the pre-mRNA (Figure 8, bottom), and consequently modifies its cellular functions. In a normal cell, this could be important to prevent deleterious splicing events in mitosis. However, in a cancer cell, this could lead to reduced apoptosis and increased proliferation.

## DATA AVAILABILITY

NMR chemical shift assignments of the Sam68 N- and C-termini have been deposited in the BioMagResBank (BMRB) under accession numbers 51359 and 51360, respectively.

MS proteomics data have been deposited in the ProteomeXchange Consortium via the PRIDE (57) partner repository with the data set identifier PXD032716.

Flow cytometry data have been deposited in the FlowRepository under accession numbers FR-FCM-Z55E (associated with Supplementary Figure S1), FR-FCM-Z55H (associated with Supplementary Figure S6) and FR-FCM-Z55Z (associated with Supplementary Figure S7).

## Supplementary Material

gkac1181_Supplemental_File
