# Supplementary material for: Cdk1-mediated threonine phosphorylation of Sam68 modulates its RNA binding, alternative splicing activity and cellular functions
Source: Nucleic Acids Res. 2022 Dec 20;50(22):13045–62. doi: 10.1093/nar/gkac1181 (PMC9825155; doi:10.1093/nar/gkac1181)
Supplement: gkac1181_Supplemental_File [file gkac1181_supplemental_file.pdf]

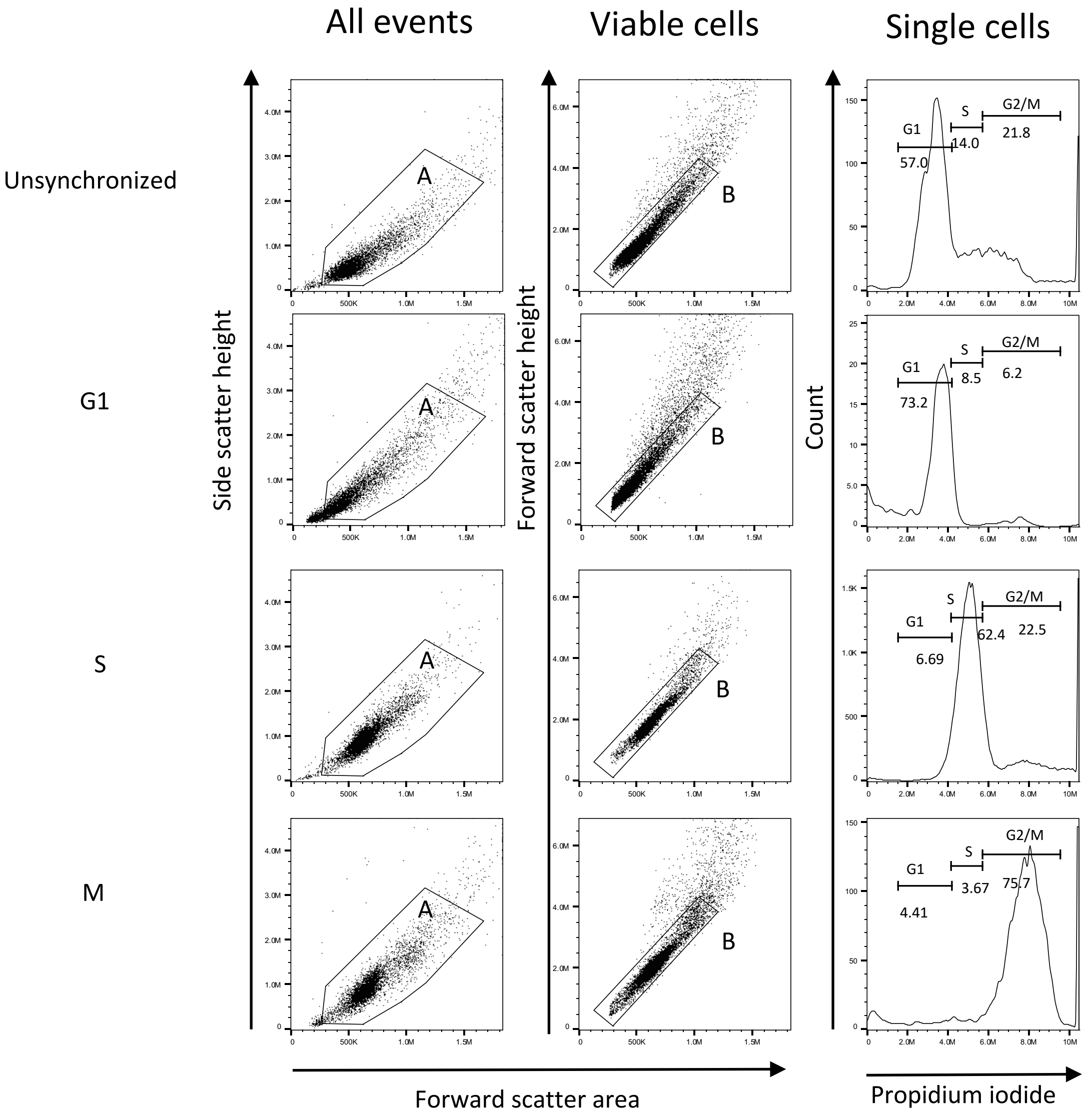

Supplementary Figure S1: Cell cycle distribution in unsynchronized and synchronized HEK293 cells.

All collected events were gated for viable cells (gate A) and single cells (gate B). Viable and single cells are gated from G1, S and G2/M phase

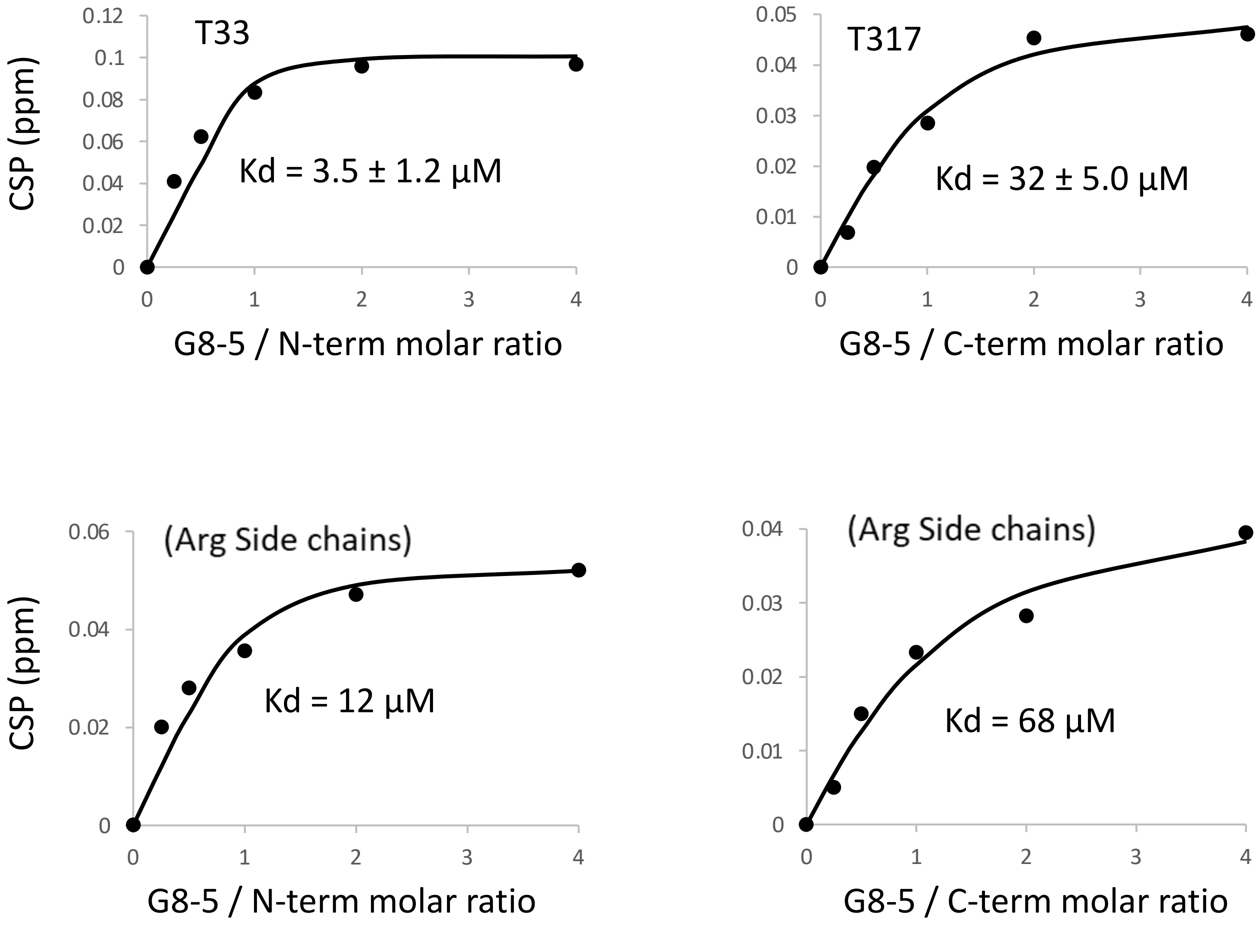

Supplementary Figure S2: Estimation of Sam68 N-term (left) and C-term (right) dissociation constants upon interaction with the G8.5 RNA.

For backbone atoms, CSP as a function of RNA/protein ratio are shown for T33 and T317 as examples. The Kd was estimated based on the CSP of eight backbone resonances. The CSP as a function of RNA/protein ratio is also displayed and the Kd estimated for arginine side chain resonances.

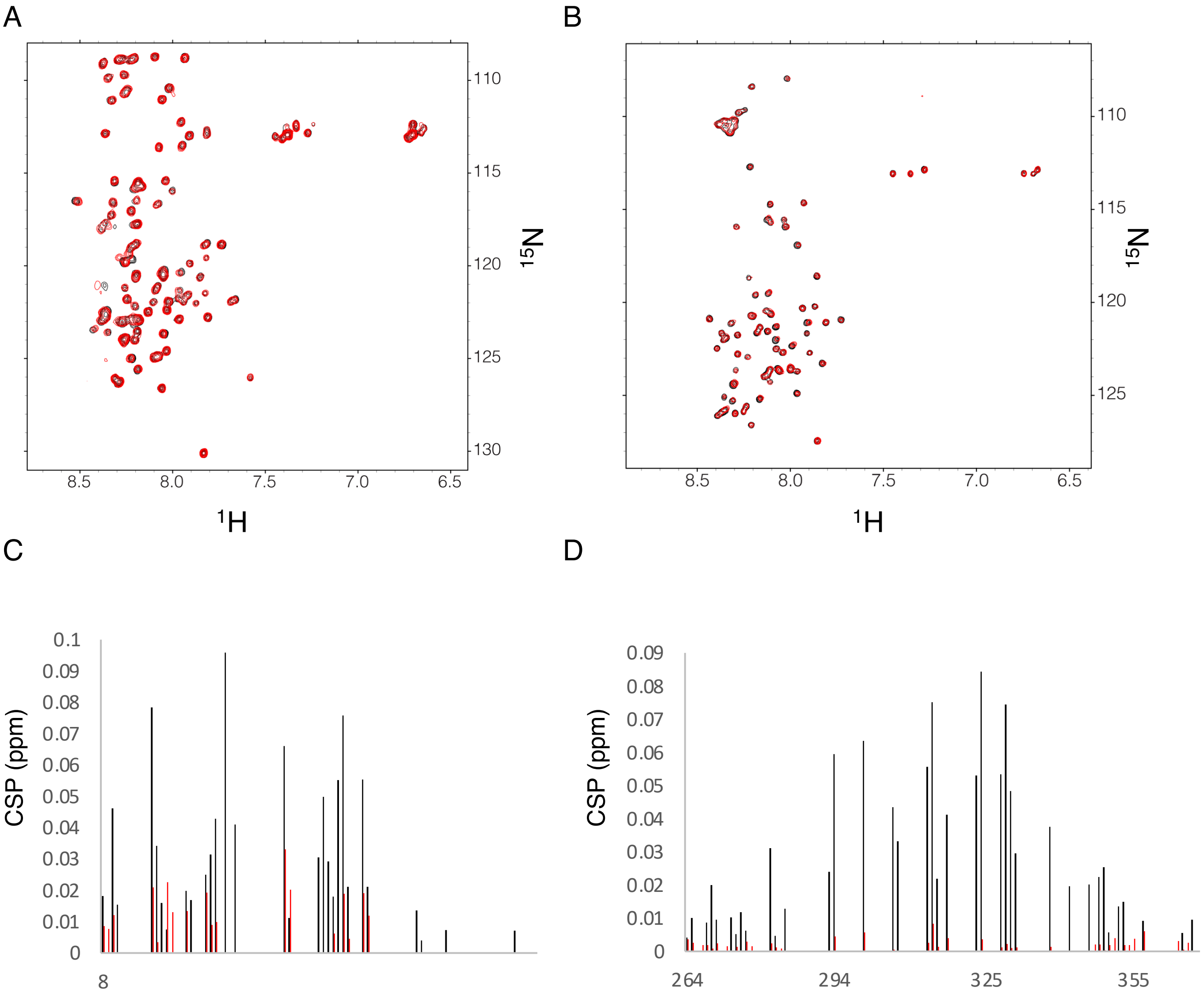

Supplementary Figure S3: NMR titration experiment of Sam68 with poly-C RNA.
(**A, B**) HSQC spectra of Sam68 N-term (A) and C-term (B) before (black) and after (red) addition of excess poly-C RNA (protein:RNA molar ratio of 1:2) at 4°C. (**C, D**) Chemical shift perturbation of Sam68 N-term (**C**) and C-term (**D**) backbone resonances upon G8.5 (black) or poly-C (red) RNAs interaction as a function of the amino acid sequence. CSPs above 0.025 (average CSP) are considered significant.

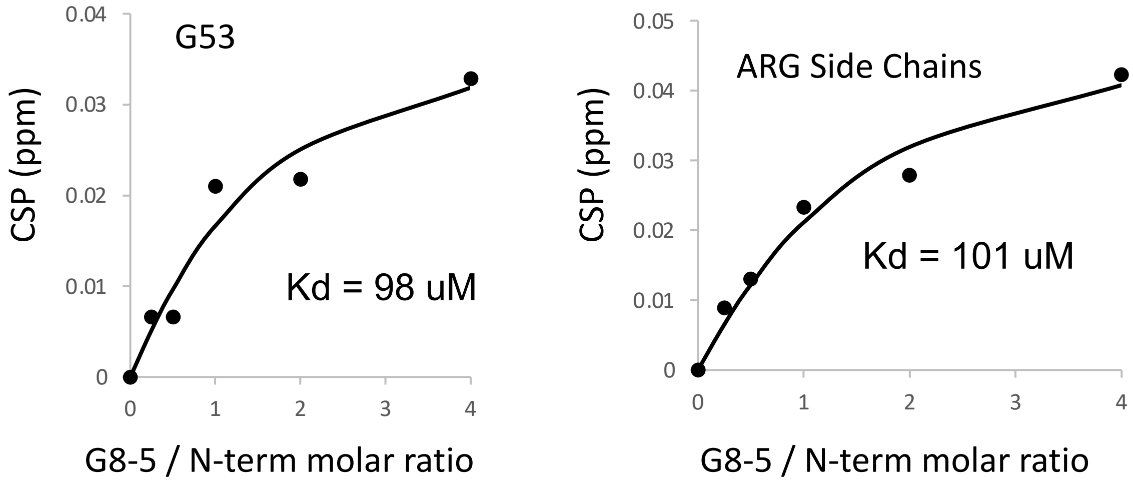

Supplementary Figure S4: Estimation of Cdk-1 phosphorylated Sam68 N-term dissociation constants upon interaction with the G8.5 RNA.

CSP as a function of RNA/protein ratio are shown for G53, and arginine side chain resonances.

A

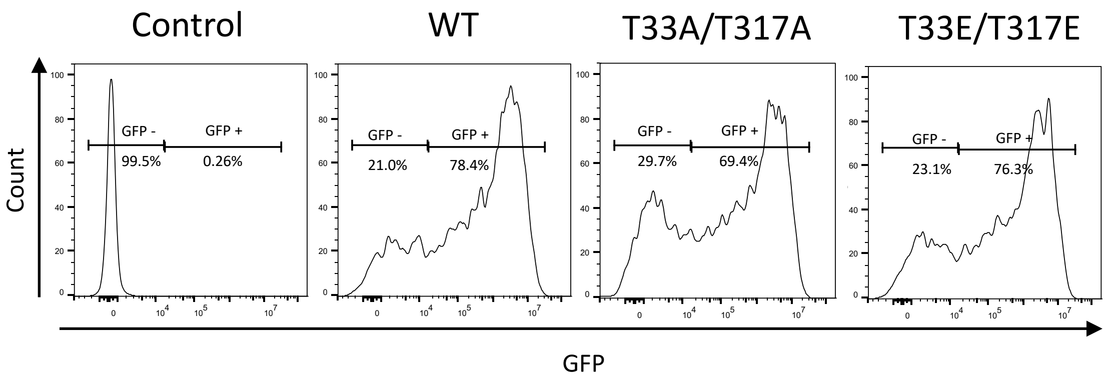

B

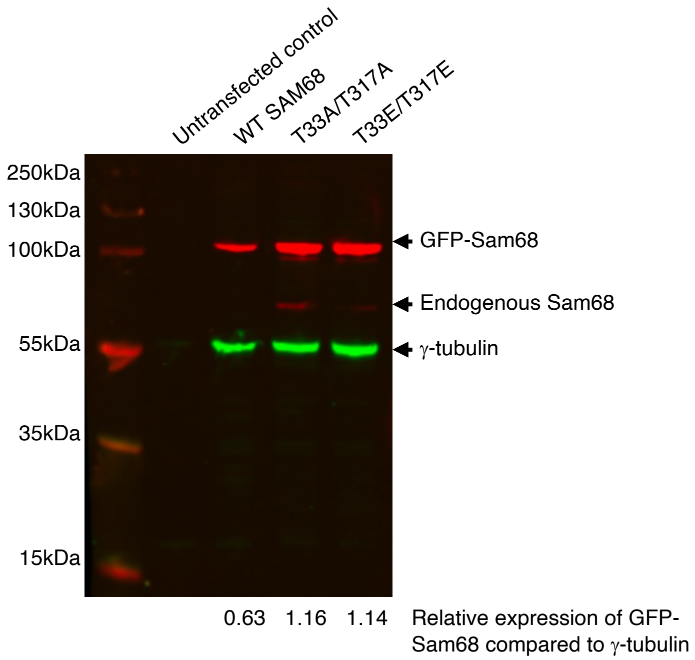

Supplementary Figure S5: **Transfection efficiency and expression levels of Sam68 WT, T33A/T317A and T33E/T317E following transfection in HCT116 cells.** (A) 48 hours after transfection of HCT116 cells with Sam68 WT or mutants, the number of GFP(+) and GFP(-) cells were counted. Cells were used for additional experiments only if more than 75% of cells were GFP positive. (B) 48 hours after transfection, cells were harvested and lysed. The cell lysate was analysed by WB using an anti-Sam68 and anti-γ-tubulin antibodies.

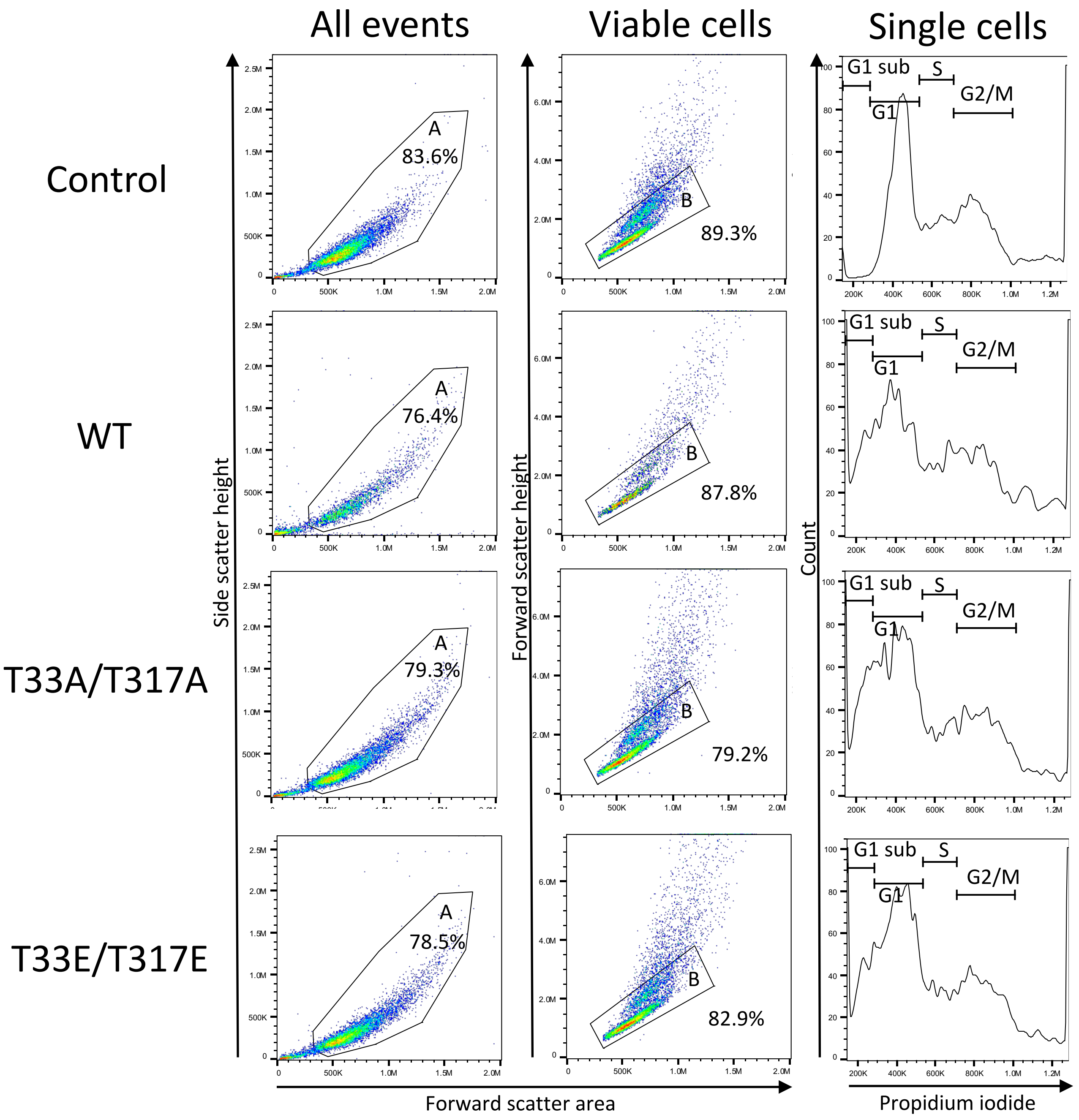

Supplementary Figure S6: Cell cycle analysis by flow cytometry
All collected events were gated for viable cells (gate A) and single cells (gate B). Viable and single cells are gated for G1 sub, G1, S and G2/M phase.

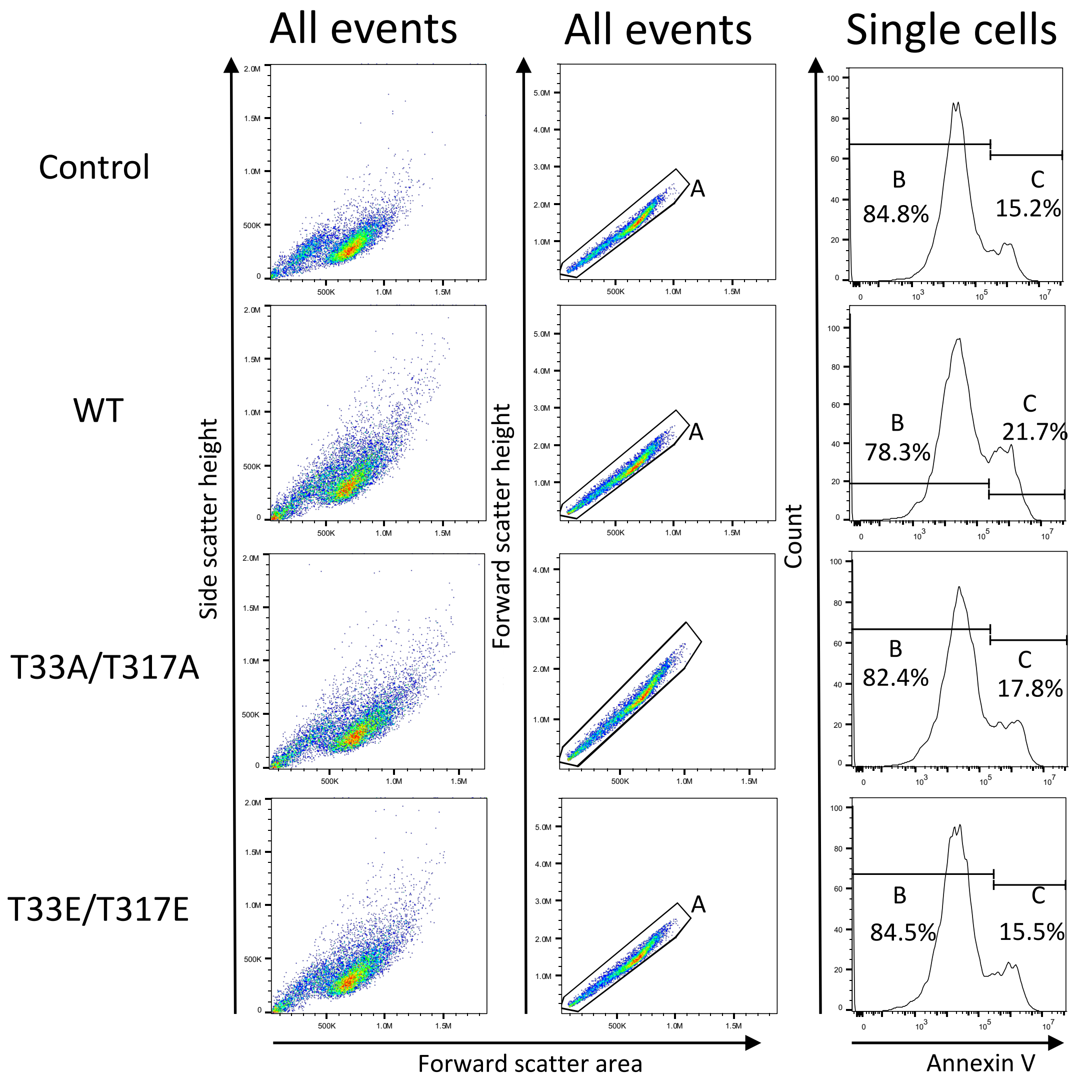

Supplementary Figure S7: Apoptosis analysis by flow cytometry

All collected events were gated for single cells (gate A). Single cells are gated for viable (gate B) and apoptotic (gate C) cells.

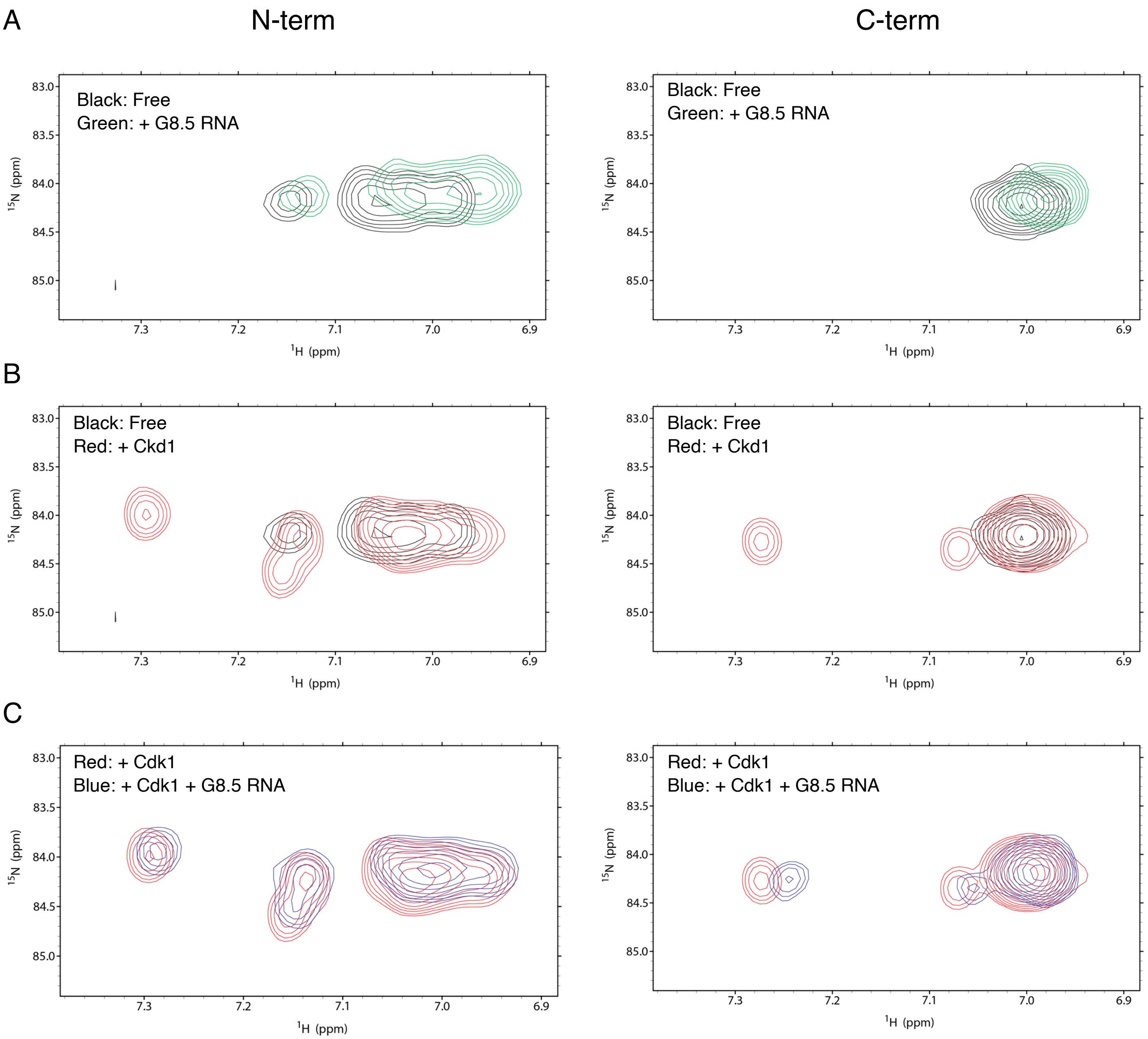

Supplementary Figure S8: NMR resonances of arginine side chains upon RNA binding or Cdk1 phosphorylation

Overlay spectra of N-term (left and C-term (right) regions of Sam68. a) resonances in the absence (black) or presence (green) of 2 molar equivalents of G8.5 RNA; b) resonances before (black) and after 8 hours incubation with Cdk1, $MgCl_2$ and ATP (red); c) resonances after Cdk1 phosphorylation in the absence (red) and presence (blue) of 2 molar equivalents of G8.5 RNA.

CD44 exon v5

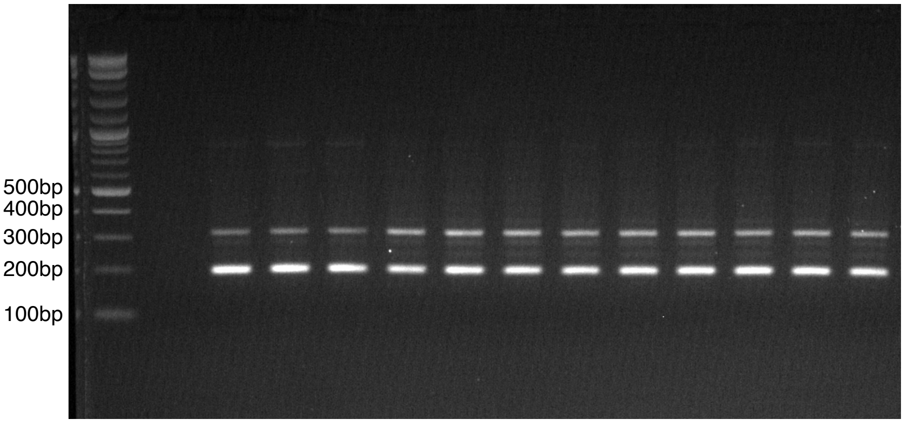

Bcl-x

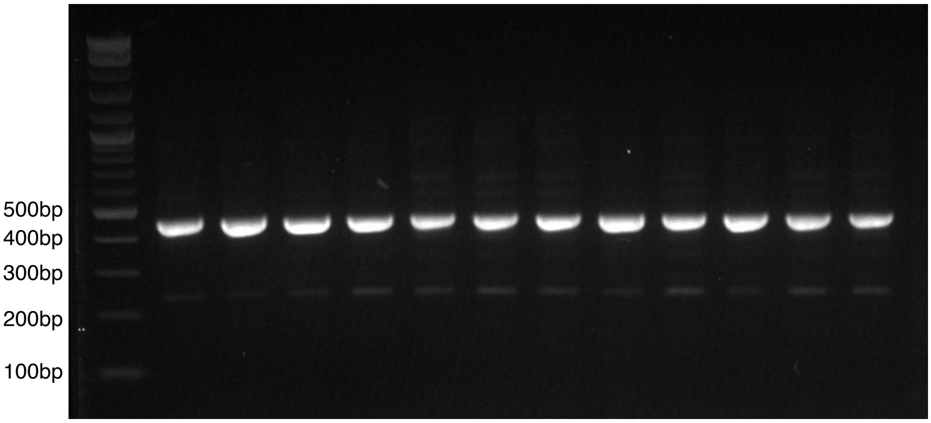

Supplementary Figure S9: uncropped gels of splicing assays 9related to Figure 6)
